# Supplementary material for: Current Practice of Physical Activity Counselling within Physiotherapy Usual Care and Influences on Its Use: A Cross-Sectional Survey
Source: Int J Environ Res Public Health. 2021 Apr 29;18(9):4762. doi: 10.3390/ijerph18094762 (PMC8125383; doi:10.3390/ijerph18094762)
Supplement: Supplementary file 1 [file ijerph-18-04762-s001.zip › ijerph-1197198-supplementary.pdf]

Supplemental File 1: Brief Physical Activity Counselling by Physiotherapists– BEHAVIOUR trial Part One: behavioural diagnosis survey

Brief Physical Activity Counselling by Physiotherapists

BEHAVIOUR trial

Part One: behavioural diagnosis questionnaire

Name: ____

Email: ____

I would like to receive a report of the study when it is completed. ☐

In this questionnaire

- ***Structured community-based physical activity*** refers to a community-based exercise, sport or physical recreation program including but not limited to fitness or community centre exercise programs, dance, tai chi, aqua aerobics, soccer, bush walking, school sport/PE, wheelchair basketball etc. These activities are done with other people (participants or instructors). They do not include home-based independent exercise programs prescribed by health professionals e.g. knee range of motion exercises, back extension exercises, sit-to-stand exercises, treadmill walking or other home-based activities such as exercise or dance to a DVD.
- ***Overall physical activity*** refers to all types of physical activity including incidental activity (e.g. walking while shopping, parking further from a destination) active transport (e.g. walking to the shops), occupational activities and household chores as well as any structured individual or group-based physical activity.
- ***Physical activity counselling*** refers to a component of patient consultation aimed at changing health behaviour as a means of primary or secondary prevention of life-style related chronic health conditions. Other benefits may also be achieved such as improvement in therapeutic health problems e.g. pain, mobility, and psychosocial outcomes. One common example is the 5A's counselling model: Assess, Advise, Agree, Assist and Arrange.

This page is intentionally blank

Demographic Information

1. Age ☐ <25 years ☐ 35-44 years ☐ 55 years and over
   ☐ 25-34 years ☐ 45-54 years

2. Gender ☐ Female ☐ Male ☐ Non-binary

3. How many years have you practised as a physiotherapist?
   ☐ 0 > 2yrs ☐ 2 ≥ 5yrs ☐ 5 ≥ 8yrs ☐ 8 ≥ 12yrs ☐ 12 ≥ 20yrs ☐ ≤20yrs

4. How many years have you worked in SWSLHD? ________________

5. Which hospital do you currently work at?
   ☐ Bankstown ☐ Bowral ☐ Braeside ☐ Fairfield
   ☐ Liverpool ☐ Campbelltown/Camden ☐ District-wide

6. Tick which boxes best match your current role in SWSLHD
   a.
   ☐ Permanent staff ☐ Contract staff ☐ First year graduate program
   b.
   ☐ Full-time ☐ Part-time ☐ Casual

   c.
   ☐ Rotational position ☐ Non-rotating position
   d.
   ☐ Level 1-2 ☐ Level 3-4 ☐ Level 5-6 ☐Manager

7. Where do you primarily see patients?
   ☐ Inpatient ☐ Outpatient ☐ Patients' homes ☐ Other______________

8. Which area of physiotherapy do you currently work in? (Please select as many as are applicable. If you have recently rotated to a new area, please fill this in related to your previous rotation area)
   ☐ Musculoskeletal ☐Rheumatology ☐Orthopaedics ☐Hands
   ☐Rehabilitation ☐Aged Care ☐Paediatrics ☐Cardiopulmonary
   ☐ED ☐Cancer ☐Medical ☐Women & Men's Health
   ☐Surgical ☐Intensive Care ☐ Other______________

9. Current workload:

a. On average, how many patients do you see in a typical work day? ______

b. On average, how long do you spend with each patient? ______ (mins)

c. On average, how many times do you see each patient before they are discharged/transferred? ________

d. On average, how many **new** patients do you see each week? ______

Readiness for structured community-based physical activity

10. Do you believe most of your patients:

	**Yes**	**No**	**Unsure**
Are ready to be referred **directly** to structured community-based physical activity programs			
Would benefit from a **transitional** stage with a health professional-led program			
Require further treatment from a health professional prior to referral to structured community- based physical activity programs			
Would benefit from a **supported introduction** or extra advice about structured community-based physical activity programs from a health professional			

Current usual care content

11. Do you think there are any interventions or assessments that you currently do in your typical usual care interactions that you could:

	**Yes**	**No**	**Unsure**
stop doing that would **not** have a negative effect on your patients?			
do more efficiently without having a negative effect on your patients?			

Current physical activity counselling in your usual healthcare interactions:

12. In a typical week, how **frequently** do you undertake each of the below for your patients who could be more active:

	**Frequently (>75% of patients)**	**Often (50-74%)**	**Sometimes (25-49%)**	**Rarely (1-24%)**	**Never**
Raise or discuss overall physical activity?					
Assess overall physical activity using a questionnaire or activity log					
Assess overall physical activity using an activity monitor or pedometer					
Set physical activity goals with your patients					
Make tailored action plans with your patients to change physical activity behaviour					
Use language to motivate physical activity behaviour change (e.g. open ended / probing questions, promoting autonomy, competence, connectedness)					
Provide a self-monitoring strategy for your patients to monitor physical activity (e.g. diary, App, pedometer)					
	**Frequently**	**Often (50-74%)**	**Sometimes (25-49%)**	**Rarely (1-24%)**	**Never**

	(>75% of patients)				
Provide handouts to your patients about physical activity					
Review physical activity status, barriers/ facilitators & provide advice in at least one subsequent session					
Investigate structured community-based physical activity opportunities for your patients					
Recommend to your patients that they attend structured community-based exercise programs e.g. group exercise, gym programs					
Recommend to your patients that they attend community-based physical recreation programs e.g. dance, tai chi, Zumba, walking groups					
Recommend to your patients that they attend community-based sport programs e.g. soccer, wheelchair basketball, swimming, athletics					
	**Frequently (>75% of patients)**	**Often (50-74%)**	**Sometimes (25-49%)**	**Rarely (1-24%)**	**Never**

Make contact with community-based exercise, physical recreation and/or sport providers regarding individual patients					
Attend ≥1 community-based exercise, physical recreation and/or sport program (e.g. individual gym program, Tai Chi or swimming) with an individual patient					

13. Which strategies have you used to identify structured community-based physical activity opportunities for your patients?

☐ None ☐ Brochures ☐ Ask other clinicians ☐ Previous patient experience

☐ Internet search ☐ In house developed list ☐ Other

____

Influences on incorporating physical activity counselling into your healthcare interactions

14. Thinking about what may influence you to incorporate physical activity counselling into your usual healthcare interactions with your patients please rate on a scale of 1 to 5 your agreement with the following statements with:
1. strongly disagree; 2. disagree; 3. neither agree or disagree (neutral); 4. agree; 5. strongly agree.

Please circle your answer

		1 (strongly disagree)		to		5 (strongly agree)
1	I have a good **knowledge** of the:					
	a. Australian physical activity guidelines for children	1	2	3	4	5
	b. Australian physical activity guidelines for adults	1	2	3	4	5
	c. Australian physical activity guidelines for older adults	1	2	3	4	5
	d. Physical activity guidelines for people with disability	1	2	3	4	5
2	I have good **knowledge** of the:					
	a. process for disability sport classification	1	2	3	4	5
	b. provisional disability sport classification	1	2	3	4	5
3	I have good **knowledge** of the:					
	a. evidence of benefit for physical activity	1	2	3	4	5
	b. evidence of harm for physical inactivity	1	2	3	4	5
	c. % people meeting physical activity guidelines	1	2	3	4	5
	d. evidence for providing physical activity counselling within healthcare	1	2	3	4	5
	e. what physiotherapists think and do about physical activity counselling within healthcare	1	2	3	4	5
	f. what patients think about health professionals bringing up physical activity	1	2	3	4	5
4	I have good **knowledge** of:					
	a. physical activity opportunities available in SWSLHD	1	2	3	4	5
	b. what to say to my patients about physical activity	1	2	3	4	5
5	I have a set routine/prompts to remember to have a conversation about physical activity within my usual healthcare interaction	1	2	3	4	5

		1 (strongly disagree)	to	5 (strongly agree)

6	I have good **skills** at:	
	a. having a conversation about physical activity within my usual healthcare interaction	1 2 3 4 5
	b. asking my patients about physical activity	1 2 3 4 5
	c. assessing physical activity subjectively	1 2 3 4 5
	d. assessing physical activity objectively	1 2 3 4 5
	e. providing brief advice about physical activity	1 2 3 4 5
	f. using motivational language with my patients	1 2 3 4 5
	g. setting physical activity goals with my patients	1 2 3 4 5
	h. setting physical activity action plans with my patients	1 2 3 4 5
	i. referring my patients to physical activity opportunities	1 2 3 4 5
	j. locating suitable physical activity opportunities available in the local community	1 2 3 4 5
	k. finding appropriate physical activity resources for my patients	1 2 3 4 5
	l. establishing which structured physical activity opportunities would be suitable for my patients	1 2 3 4 5
	m. making referrals to suitable structured community-based physical activity opportunities	1 2 3 4 5

		1 to 5<br>(strongly disagree) (strongly agree)				
7	I **believe**:					
	a. There are too many other important things to do in my healthcare interaction than discuss physical activity	1	2	3	4	5
	b. Physical activity counselling is not a part of a physiotherapists job description	1	2	3	4	5
	c. Participating in physical activity is not usually one of my patients' goals	1	2	3	4	5
	d. My patients' are resistant to discuss physical activity	1	2	3	4	5
	e. Providing physical activity counselling will not change my patient's physical activity levels	1	2	3	4	5
	f. Community physical activity providers are unlikely to be able to cater appropriately and safely for my patients	1	2	3	4	5
8	I **feel**:					
	a. physical activity counselling is hard to do and not worth it	1	2	3	4	5
	b. frustrated to try to fit in physical activity counselling as it takes away from time on the therapeutic problem	1	2	3	4	5
	c. my patients don't really want me to talk to them about physical activity	1	2	3	4	5
	d. worried there may be a negative outcome for my patients if I refer them to a community physical activity opportunity	1	2	3	4	5
	e. my patient/family/carer is already too overburdened with their situation to increase extra demands on them	1	2	3	4	5
	f. not confident in the capacity of structured physical activity leaders to manage my client	1	2	3	4	5
		1 to 5<br>(strongly disagree) (strongly agree)				

9	I have a set routine/process for conducting an assessment/intervention session with my patients	1	2	3	4	5
10	My time is too limited to:					
	a. include physical activity counselling	1	2	3	4	5
	b. locate suitable structured community-based physical activity opportunities	1	2	3	4	5
	c. make referrals to suitable structured physical activity opportunities	1	2	3	4	5
11	I have found none or limited options suitable for my patients in the local area	1	2	3	4	5
12	My patients do not have:					
	a. access to the funding to enable participation in paid community physical activities	1	2	3	4	5
	b. access to transport to enable participation	1	2	3	4	5
	c. family/carers support to participate (e.g. competing family responsibilities, lack of money, lack of time or transport issues)	1	2	3	4	5
13	Providing physical activity advice to patients:					
	a. is common in my immediate team	1	2	3	4	5
	b. is common in my department	1	2	3	4	5
	c. is seen as a priority in my hospital	1	2	3	4	5
	d. is seen as a priority in SWSLHD	1	2	3	4	5
	e. is seen as a priority in my profession	1	2	3	4	5
**Are there any other influencers you would like to tell us about?**						

Training

15. Have you attended training by HealthChange Australia? ☐ No ☐ Yes (If yes, which year)________

16. Have you attended or completed any other relevant courses on behaviour change/ motivational interviewing/ health coaching? □ No □ Yes

If yes, please provide details

____

17. Do you have any other comments?

____

____

Thank you for completing this questionnaire!

Supplemental File 2: Influences on incorporating physical activity counselling into physiotherapy routine healthcare (n=84)

Perceived barriers	Strongly disagree	Disagree	Neither agree or disagree	Agree	Strongly agree	% Reported barriers[#]
**Psychological Capabilities: (knowledge) [0-14]**						
**I have good knowledge of**						
Australian physical activity guidelines for adults	1%	1%	8%	43%	46%	10%
Australian physical activity guidelines for older adults	2%	6%	16%	53%	23%	24%
Australian physical activity guidelines for children	9%	32%	27%	28%	5%	68%
physical activity guidelines for people with disability	16%	28%	33%	17%	6%	77%
**I have good knowledge of**						
process for disability sport classification	55%	37%	5%	4%	0%	97%
provisional disability sport classification	56%	36%	6%	2%	0%	98%
**I have good knowledge of**						
evidence of benefit for physical activity	0%	0%	2%	40%	57%	2%
evidence of harm for physical inactivity	0%	5%	12%	40%	43%	17%
% people meeting physical activity guidelines	11%	33%	31%	20%	5%	75%
evidence for providing physical activity counselling within healthcare	2%	27%	33%	30%	7%	62%
what physiotherapists think and do about physical activity counselling within healthcare	5%	29%	37%	27%	2%	71%
what patients think about health professionals bringing up physical activity	5%	37%	37%	20%	1%	79%
**I have good knowledge of**						
physical activity opportunities available in SWSLHD	2%	20%	39%	36%	2%	61%
what to say to my patients about physical activity	0%	5%	20%	62%	13%	25%
**Psychological Capability: (self-regulation) [0-1]**						
I have a set routine/prompts to remember to have a conversation about physical activity within my usual healthcare interaction	6%	32%	26%	23%	13%	64%

Perceived barriers	Strongly disagree	Disagree	Neither agree or disagree	Agree	Strongly agree	% Reported barriers[#]
**Psychological Capability: (cognition) [0-13]**						
**I have good skills delivering these elements of physical activity counselling**						
having a conversation about physical activity within my usual healthcare interaction	0%	6%	21%	57%	15%	27%
asking my patients about physical activity	0%	2%	17%	55%	26%	19%
assessing physical activity subjectively	0%	5%	17%	57%	21%	22%
assessing physical activity objectively	2%	25%	27%	31%	14%	54%
providing brief advice about physical activity	0%	2%	8%	63%	26%	10%
using motivational language with my patients	1%	8%	26%	50%	14%	35%
setting physical activity goals with my patients	0%	4%	18%	67%	12%	22%
setting physical activity action plans with my patients	1%	17%	35%	36%	11%	53%
referring my patients to physical activity opportunities	0%	19%	24%	45%	12%	43%
locating suitable physical activity opportunities available in the local community	2%	26%	25%	38%	8%	53%
finding appropriate physical activity resources for my patients	2%	20%	33%	36%	8%	55%
establishing which structured physical activity opportunities would be suitable for my patients	4%	10%	36%	46%	5%	50%
making referrals to suitable structured community-based physical activity opportunities	5%	18%	27%	39%	11%	50%
**Motivation (reflective): [0-6]**						
**I believe**						
There are too many other important things to do in my healthcare interaction than discuss physical activity*	43%	33%	19%	5%	0%	24%
Physical activity counselling is not a part of a physiotherapists job description*	65%	29%	5%	1%	0%	6%

Perceived barriers	Strongly disagree	Disagree	Neither agree or disagree	Agree	Strongly agree	% Reported barriers#
Participating in physical activity is not usually one of my patients' goals*	27%	27%	18%	22%	7%	47%
My patients are resistant to discuss PA*	6%	34%	40%	20%	0%	60%
Providing physical activity counselling will not change my patient's physical activity levels*	28%	41%	25%	6%	0%	31%
Community physical activity providers are unlikely to be able to cater appropriately and safely for my patients*	18%	31%	31%	17%	2%	50%
**Motivation (automatic): wants/feelings [0-6]**						
**I feel**						
physical activity counselling is hard to do and not worth it*	40%	46%	14%	0%	0%	14%
frustrated to try to fit in physical activity counselling as it takes away from time on the therapeutic problem*	32%	40%	17%	10%	1%	28%
my patients don't really want me to talk to them about physical activity*	12%	32%	35%	21%	0%	56%
my patient/family/carer is already too overburdened with their situation to increase extra demands on them*	5%	34%	33%	23%	6%	62%
worried there may be a negative outcome for my patients if I refer them to a community physical activity opportunity*	34%	48%	11%	7%	0%	18%
not confident in the capacity of structured physical activity leaders to manage my patient*	10%	40%	30%	18%	2%	50%
**Motivation (automatic): urges/habits [0-1]**						
I have a set routine/process for conducting an assessment/intervention session with my patients	2%	12%	20%	45%	21%	34%
**Opportunity (Physical) [0-7]**						
**My time is too limited to:**						
include physical activity counselling*	20%	44%	24%	11%	1%	36%

Perceived barriers	Strongly disagree	Disagree	Neither agree or disagree	Agree	Strongly agree	% Reported barriers#
locate suitable structured community-based physical activity opportunities*	13%	29%	23%	30%	4%	57%
make referrals to suitable structured physical activity opportunities*	18%	45%	21%	16%	0%	37%
**My patients do not have**						
funding for participation*	0%	5%	24%	45%	26%	95%
transport*	0%	7%	16%	51%	26%	93%
support*	1%	6%	17%	55%	21%	93%
**Opportunity (Social) [0-5]**						
**Providing physical activity advice to patients:**						
is seen as a priority in my profession	0%	5%	18%	49%	28%	23%
is common in my immediate team	2%	9%	24%	56%	9%	35%
is common in my department	2%	11%	28%	49%	10%	41%
is seen as a priority in SWSLHD	2%	20%	49%	22%	7%	71%
is seen as a priority in my hospital	4%	23%	45%	23%	5%	72%

Key: # Barriers determined as percentage of physiotherapists who selected strongly disagree, disagree or neither agree or disagree. *For these influencers, barriers determined as percentage of physiotherapists who selected strongly agree, agree, or neither agree or disagree.

#
#

Supplemental File 3: Relationship between self-reported skills and use of different PA counselling elements within usual physiotherapy care

I have good skills at		PA counselling elements		Total, n	Relative Risk (95% CI)
		I raise or discuss overall PA to >50% patients, n (row%)	I raise or discuss overall PA to <50% patients, n (row%)		
having a conversation about PA within my usual healthcare interaction	Yes	48 (79)	13 (21)	61	1.6 (1.1 to 2.6)*
	No	11 (48)	12 (52)	23	
asking my patients about physical activity	Yes	53 (79)	14 (21)	67	2.2 (1.2 to 4.3)*
	No	6 (35)	11 (65)	17	
providing brief advice about physical activity	Yes	55 (74)	19 (26)	74	1.9 (0.9 to 4.0)
	No	4 (40)	6 (60)	10	
		I assess overall physical activity subjectively to > 50% patients, n (row%)	I assess overall physical activity subjectively to < 50% patients, n (row%)		
assessing physical activity subjectively	Yes	7 (11)	58 (89)	65	2.0 (0.3 to 15.6)
	No	1 (5)	18 (95)	19	
		I assess overall physical activity objectively to > 50% patients, n (row%)	I assess overall physical activity objectively to < 50% patients, n (row%)		
assessing physical activity objectively[#]	Yes	0 (0)	38 (100)	38	—
	No	0 (0)	46 (100)	46	
		I set physical activity goals to > 50% patients, n (row%)	I set physical activity goals to < 50% patients, n (row%)		
setting physical activity goals with my patients	Yes	43 (66)	22 (34)	65	3.1 (1.3 to 7.6)*
	No	4 (21)	15 (79)	19	
		I make tailored action plans to change physical activity behavior to > 50% patients, n (row%)	I make tailored action plans to change physical activity behavior to < 50% patients, n (row%)		
setting physical activity action plans with my patients	Yes	24 (61)	15 (39)	39	2.1 (1.2 to 3.5)*
	No	13 (30)	31 (71)	44	
**I have good skills at**		**PA counselling elements**		**Total, n**	**Relative Risk (95% CI)**
		I use language to motivate physical activity behavior change to > 50% patients, n (row%)	I use language to motivate physical activity behavior change to < 50% patients, n (row%)		
using motivational language with my patients	Yes	40 (75)	13 (25)	53	2.1 (1.3 to 3.5)*
	No	11 (36)	20 (65)	31	

		I provide handouts about physical activity to > 50% patients, n (row%)	I provide handouts about physical activity to < 50% patients, n (row%)		
finding appropriate physical activity resources for my patients	Yes	21 (57)	16 (43)	37	1.7 (1.0 to 2.7)
	No	16 (34)	31 (66)	47	
		I investigate structured community-based physical activity opportunities to > 50% patients, n (row%)	I investigate structured community-based physical activity opportunities to < 50% patients, n (row%)		
locating suitable physical activity opportunities available in the local community	Yes	19 (49)	20 (51)	39	1.9 (1.1 to 3.6)*
	No	11 (25)	33 (75)	44	
		I recommend to attend structured community-based exercise to > 50% patients, n (row%)	I recommend to attend structured community-based exercise to < 50% patients, n (row%)		
referring my patients to physical activity opportunities	Yes	33 (70)	14 (30)	47	3.2 (1.7 to 6.2)*
	No	8 (21)	29 (78)	37	
		I make contact with PA providers to > 50% patients, n (row%)	I make contact with PA providers to < 50% patients, n (row%)		
making referrals to suitable structured community-based physical activity opportunities#	Yes	8 (19)	34 (81)	42	—
	No	0 (0)	42 (100)	42	

Key: *statistically significant p<0.05; #Due to an empty cell, no statistics were computed for having good skills at assessing physical activity objectively, making referrals to suitable structured community-based physical activity opportunities and their corresponding PA counselling elements.

Footnote: If relative risk and 95%CI were >1, this indicates that those who reported having the specific skill were significantly more likely to report using the corresponding physical activity element in routine practice.
